# Polyhedral Palladium–Silver Alloy Nanocrystals as Highly Active and Stable Electrocatalysts for the Formic Acid Oxidation Reaction

**DOI:** 10.1038/srep13703

**Published:** 2015-09-02

**Authors:** Geng-Tao Fu, Chang Liu, Qi Zhang, Yu Chen, Ya-Wen Tang

**Affiliations:** 1 Jiangsu Key Laboratory of New Power Batteries, Jiangsu Collaborative Innovation Center of Biomedical Functional Materials, School of Chemistry and Materials Science, Nanjing Normal University, Nanjing 210023, P. R. China; 2 Key Laboratory of Macromolecular Science of Shaanxi Province, School of Materials Science and Engineering, Shaanxi Normal University, Xi’an 710062, P. R. China

## Abstract

Polyhedral noble–metal nanocrystals have received much attention and wide applications as electrical and optical devices as well as catalysts. In this work, a straightforward and effective hydrothermal route for the controllable synthesis of the high-quality Pd–Ag alloy polyhedrons with uniform size is presented. The morphology, composition and structure of the Pd–Ag alloy polyhedrons are fully characterized by the various physical techniques, demonstrating the Pd–Ag alloy polyhedrons are highly alloying. The formation/growth mechanisms of the Pd–Ag alloy polyhedrons are explored and discussed based on the experimental observations and discussions. As a preliminary electrochemical application, the Pd–Ag alloy polyhedrons are applied in the formic acid oxidation reaction, which shows higher electrocatalytic activity and stability than commercially available Pd black due to the “synergistic effects” between Pd and Ag atoms.

Direct formic acid fuel cells (DFAFCs), a friendly electrochemical energy conversion device, has received widely attention for portable electronics owing to the high efficiency, high specific energy density and low pollution^1^,^2^,^3^,^4^,^5^,^6^,^7^,^8^,^9^,^10^. The performance of DFAFCs is largely determined by the anodic electrocatalysts for the formic acid oxidation reaction (FAOR). Thus, the design and synthesis of the highly active anodic electrocatalysts is an important and active research topic. Although Pt-based nanocrystals have been recognized as the promising electrocatalysts for the FAOR due to the remarkably inherent chemical stability of Pt in acidic medium, many hurdles have to be eliminated before the large-scale commercialization, including the low storage and high cost of Pt, together with a poor CO_ads_ intermediate tolerance due to the strong Pt–CO bond^10^,^11^,^12^,^13^. Compared to Pt-based electrocatalysts, Pd-based electrocatalysts have recently drawn considerable attention because they effectively catalyze FAOR via a direct dehydrogenation pathway, which reduces the CO_ads_ intermediate poisoning.

Alloying Pd with other metals M (M = Ni, Co, Cu, Ag, Au, etc.) can dramatically improve the electrocatalytic activity and durability of Pd nanocrystals through modifying electronic structure and geometrical structure of Pd atoms^7^,^14^,^15^,^16^,^17^. For example, Xing and co-workers demonstrated that the Pd–Ni_2_P/C electrocatalyst exhibited the improved electrocatalytic activity and stability for the FAOR compared to that of the commercial Pd black due to the strong electronic interaction between Pd and Ni_2_P^7^. Among various Pd–M alloy nanocrystals, the Pd–Ag alloy nanocrystals generally exhibit the enhanced electrocatalytic ability and resistance to deactivation due to the highly synergistic interaction between Pd and Ag^14^,^15^. According to Hammer–Nørskov’s calculation^18^, the d-band center of Pd with a lattice value of 3.89 Å will obviously up-shift when alloying Pd with Ag with a lattice value of 4.09 Å^19^. Meanwhile, the introduction of Ag can accelerate the oxidation of the poisonous intermediates and consequently inhibits the poisoning of the Pd active sites^12^.

To date, the galvanic replacement reaction provides an effective route for generating the bimetallic Pd–Ag nanocrystals with different morphology and surface arrangement by using Ag nanocrystals as sacrificial templates^20^,^21^,^22^,^23^,^24^,^25^. For instance, Xia and co-workers prepared the Pd–Ag alloy nanoboxes using Ag nanocubes as sacrificial templates^20^. More recently, the similar approach was also used to synthesize the triangular Ag–Pd alloy nanoplates^21^. Although the two-step galvanic replacement reaction can successfully synthesize the Pd–Ag alloy nanocrystals, the synthesis route suffers from the complex reaction steps, extensive operator skills and even relatively high costs. Thus, a facile one-pot chemical reduction method for the synthesis of the Pd–Ag alloy nanocrystals is extremely desirable. More recently, Xu and co-workers have developed a non-noble metal sacrificial approach to successfully synthesize the reduced graphene oxide (RGO) supported PdAg nanocrystals, which opens up a new avenue for the development of high-performance PdAg nanocatalysts^26^.

In this work, we present a straightforward and effective hydrothermal route for the synthesis of the high-quality Pd–Ag alloy polyhedrons. Based on the experimental observations, the formation of the Pd–Ag alloy polyhedrons mainly originates from the underpotential deposition (UPD)-based epiphytic reduction mechanism. The as-prepared Pd–Ag alloy polyhedrons show the enhanced electrocatalytic activity and durability for the FAOR compared to commercial Pd black.

## Results

### Physicochemical characterization of the Pd–Ag alloy polyhedrons

**Figure 1:**
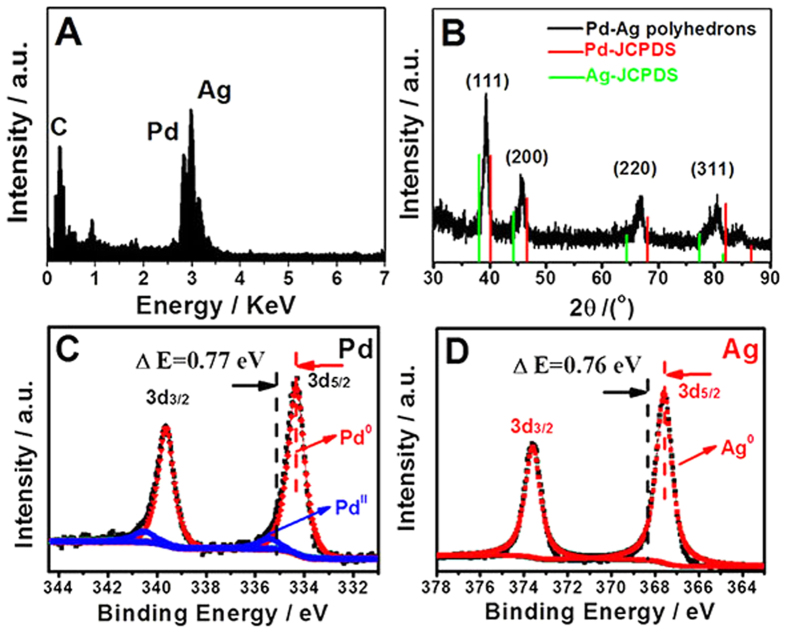
(**A**) EDX spectrum, (**B**) XRD pattern, (**C**) Pd 3d XPS spectrum, and (**D**) Ag 3d XPS spectrum of the Pd–Ag alloy polyhedrons. (Note: the vertical black dotted lines in (**C**) and (**D**) represent the standard values of Pd3d 5/2 and Ag3d 5/2, respectively).

In a typical synthesis, the Pd–Ag alloy polyhedrons were obtained by reducing K_2_PdCl_4_ and AgNO_3_ precursors with HCHO in polyallylamine hydrochloride (PAH, Scheme S1) aqueous solution at 180 °C for 2 h (see Experimental section for details). The chemical compositions, crystal structures, and chemical states of the products were first analyzed by energy dispersive X-ray (EDX), X-ray diffraction (XRD), and X-ray photoelectron spectroscopy (XPS) spectra, respectively. EDX analysis shows the products contain both Pd and Ag elements, and the elemental compositions of Pd and Ag are 48.10 and 51.90 at%, in accordance with theoretical stoichiometric proportion (Fig. 1A). The XRD pattern of the Pd–Ag nanocrystals clearly shows the four different diffraction peaks, which can be indexed to (111), (200), (220), and (311) facets of face-centered-cubic (*fcc*) metal, respectively (Fig. 1B). Meanwhile, no apparent diffraction peaks of pure Pd or Ag are detected, and all diffraction peaks of the Pd–Ag nanocrystals are located between those of Pd (JCPDS-46-1043) and Ag (JCPDS-04-0783), suggesting the formation of the Pd–Ag alloy. The average lattice constant (*a*) of the Pd–Ag alloy nanocrystals calculated from the four different diffraction peaks is 0.39911 nm, which is bigger than standard value (0.38902 nm)^27^,^28^ of *fcc* Pd (JCPDS-46–1043). The lattice expansion originates from the partial replacement of Pd atoms by Ag atoms with a larger atom radius (Pd: 0.128 nm vs. Ag: 0.134 nm). Based on the Vegard’s law^29^, it can be deduced from the XRD pattern that the ratio of Pd/Ag was approximately 1:1, in accordance with the EDX analysis.

XPS, a highly surface sensitive technique, was performed to investigate the chemical states of the surface elements. XPS measurement shows the Pd/Ag atomic ratio is ca. 0.953, corresponding to 48.80% and 51.20% of the Pd and Ag, respectively (Figure S1). This value is highly consistent with the bulk composition obtained by EDX and XRD, further confirming the formation of the Pd–Ag alloy. Meanwhile, Pd 3d and Ag 3d XPS spectra display two peaks at 3d5/2 and 3d3/2 positions with a spin-orbit separation of 5.26 and 6.0 eV, respectively, which confirms the generation of metallic Pd and Ag (Fig. 1C,D). In particular, the Pd 3d (3d5/2 = 334.33 eV) and Ag 3d (3d5/2 = 367.54 eV) binding energies of the Pd–Ag alloy nanocrystals are negatively shifted ca. 0.77 eV and 0.76 eV compared to the standard values of bulk Pd (3d5/2 = 335.10 eV) and Ag (3d5/2 = 368.30 eV), respectively, attributing to the interaction between residual PAH and metal atoms^30^,^31^. Due to the formation of N-Pd bond, the lone pair electrons of –NH_2_ groups in PAH effectively donate electrons to Pd, resulting in the shift of elemental binding energy.

**Figure 2:**
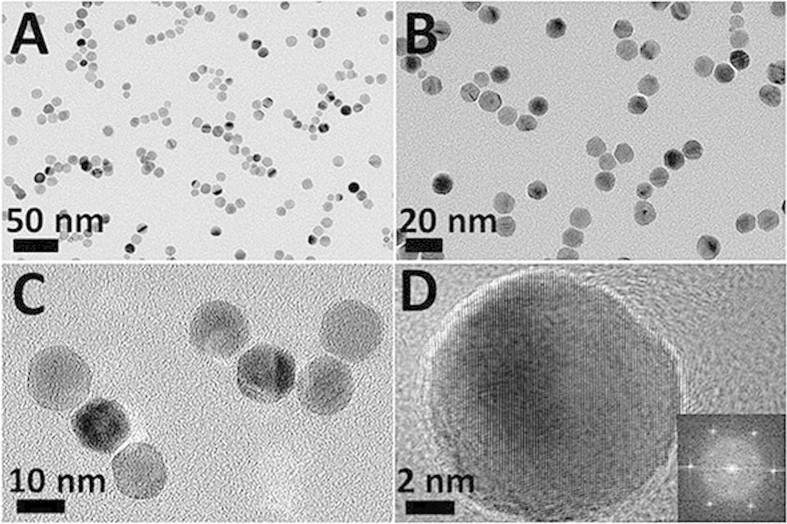
(**A**,**B**) Typical TEM images and (**C**) HRTEM image of the Pd–Ag alloy polyhedrons. (**D**) HRTEM image of an individual Pd–Ag polyhedron, the inset show the corresponding FFT pattern.

The morphology of the Pd–Ag alloy nanocrystals was investigated using transmission electron microscopy (TEM). As observed in low-resolution TEM images, the as-synthesized Pd–Ag alloy nanocrystals exhibit the like-sphere morphology in terms of size and distribution (Fig. 2A,B and Figure S2). The average size of the Pd–Ag alloy nanocrystals is ca. 12 ± 3 nm. In fact, the Pd–Ag alloy nanocrystals are polyhedral nanostructures with obvious edges and borders, which can be observed on the high-resolution TEM (HRTEM) (Fig. 2C). The fast Fourier transform (FFT) pattern on an individual Pd–Ag alloy nanocrystals clearly demonstrates the single-crystalline nature (Fig. 2D). The interval between two lattice fringes from the magnified HRTEM image is measured to be 0.228 nm (Figure S3), closed to the {111} lattice spacing at this crystal orientations.

**Figure 3:**
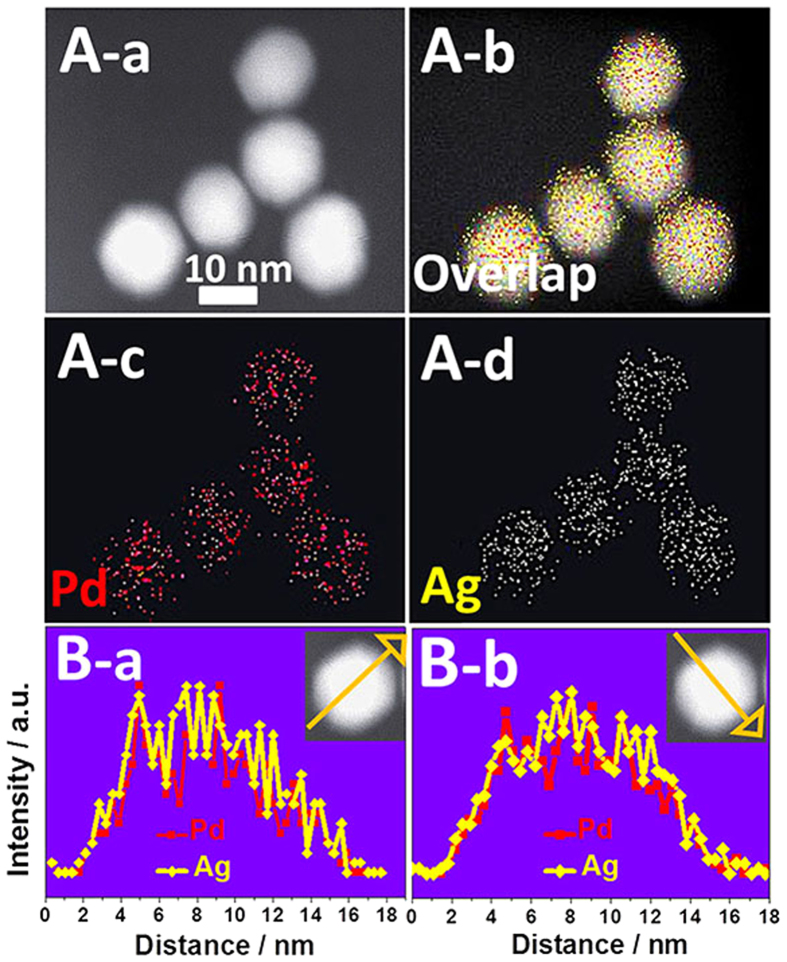
(**A**) HAADF-STEM and EDX elemental mapping images of the Pd–Ag alloy polyhedrons. (**B**) EDX line scanning profiles of an individual Pd–Ag polyhedron.

The positional distribution of Pd and Ag in the Pd–Ag alloy polyhedrons was revealed by high-angle annular dark-field scanning TEM (HAADF-STEM), EDX elemental mapping pattern and EDX line scanning profile. The HAADF-STEM image reveals the same luminance through the whole polyhedrons (Fig. 3A-a), suggesting that the Pt–Ag polyhedrons have an alloy structure rather than a core-shell structure^31^,^32^. EDX elemental mapping pattern (Fig. 3A) and EDX line scanning profile (Fig. 3B) of Pd and Ag unambiguously verify that the distribution of Pd and Ag completely overlaps.

**Figure 4:**
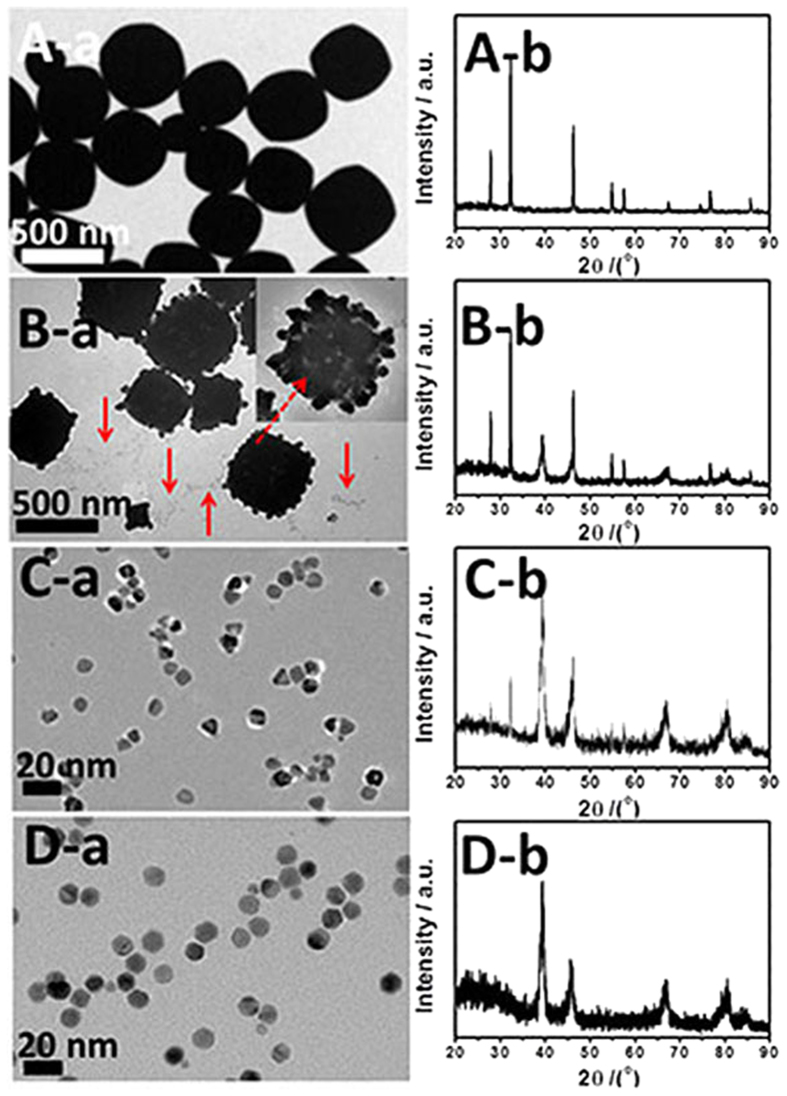
TEM images and XRD patterns of the intermediates collected at different growth stages: (A) 0 min, (B) 30 min, (C) 1 h and (D) 2 h.

To better understand the formation/growth process of the Pd–Ag alloy polyhedrons, the intermediate nanocrystals produced at different reaction stages were investigated by TEM and XRD. At the initial stage of the reaction (0 min), due to the presence of an excess amount of Cl^–^, white like-cube AgCl precipitate is generated instantaneously when AgNO_3_ is introduced in the reaction system (Fig. 4A-a), which is confirmed by XRD pattern of white precipitate (Fig. 4A-b). At 30 min, AgCl nanocubes evolve into the porous AgCl nanocrystals (Fig. 4B-a), because Ag^+^ ions gradually liberate from AgCl with the increase of reaction temperature^33^. Meanwhile, it is found that there are many tiny Pd–Ag alloy nanocrystals with different morphology (e.g., cubes, triangular plates and spheres) around the porous AgCl nanocubes, as indicated by the magnified TEM image and EDX line scanning profiles (Figure S4). Furthermore, XRD pattern of the intermediate nanocrystals at 30 min (Fig. 4B-b) also confirms that AgCl nanocrystals and the Pd–Ag alloy nanocrystals are coexistent in the reaction system. With an increase of reaction time to 1 h, the AgCl nanocrystals with lager size disappear, accompanying with the generation of the different-shaped Pd–Ag nanocrystals (Fig. 4C). As the reaction proceeded to 2 h, the complete Pd–Ag alloy polyhedrons generate (Fig. 4D). The shape evolution of the Pd–Ag alloy polyhedrons is also reflected in the color change of the reaction solution (Figure S5). The color of the solution changed from grey white to dark brown, and finally to black over the course of reaction, which corresponds to the sequential dissolution of AgCl precipitate and the formation of the Pd–Ag alloy polyhedrons.

### Electrocatalytic tests

**Figure 5:**
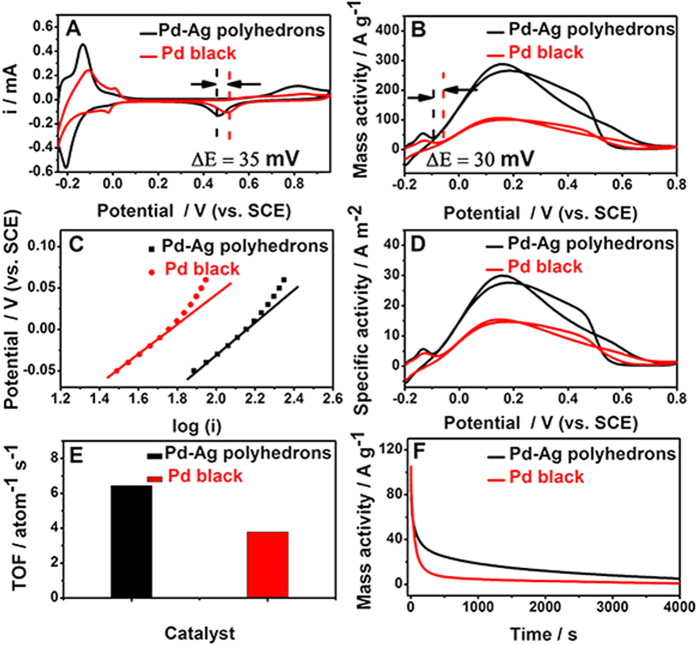
(**A**) Cyclic voltammograms of the Pd–Ag alloy polyhedrons and commercial Pd black in N_2_-saturated 0.5 M H_2_SO_4_ solution at a scan rate of 50 mV s^−1^. (**B**,**D**) Mass-normalized and ECSA-normalized cyclic voltammograms of the Pd–Ag alloy polyhedrons and commercial Pd black in N_2_-saturated 0.5 M H_2_SO_4_ + 0.5 M HCOOH solution at a scan rate of 50 mV s^−1^, respectively. (**C**) Tafel plots of log I vs. potential for the FAOR on the Pd–Ag alloy polyhedrons and commercial Pd black in electrochemical control area. (**E**) The TOF values of the Pd–Ag alloy polyhedrons and commercial Pd black at 0.15 V potential. (**F**) Chronoamperometry curves of the Pd–Ag alloy polyhedrons and commercial Pd black in N_2_-saturated 0.5 M H_2_SO_4_ + 0.5 M HCOOH solution for 4000 s at 0.15 V potential.

The electrocatalytic activity of the Pd–Ag alloy polyhedrons for the FAOR was evaluated in an electrochemical measurement system. For comparison, the commercial state-of-the-art Pd black was also measured as reference materials under the same conditions. The electrochemical properties of the Pd–Ag alloy polyhedrons and commercial Pd black are investigated by cyclic voltammetry in N_2_-purged 0.5 M H_2_SO_4_ solution (Fig. 5A). No electrochemical dissolution of Ag is found, indicating that alloying with Pd can greatly enhance the electrochemical stability of Ag. In addition, after an additional 10 CV cycles, the Pd/Ag atom ratio (50.30: 49.70) in Pd–Ag alloy polyhedrons is close to their initial value (48.10 and 51.90) (Figure S6A), and the the morphology of Pd–Ag alloy polyhedrons essentially remains (Figure S6B). The results further confirm that the introduction of Pd enhances the electrochemical stability of Ag. The hydrogen adsorption peak of the Pd–Ag alloy polyhedrons is much larger than that of the commercial Pd black, indicating that the Pd–Ag alloy polyhedrons may have a bigger electrochemically active surface area (ECSA) than Pd black. Based on the charge of reduction monolayer in Pd oxide region (see Experimental section for details), the ECSA of the Pd–Ag alloy polyhedrons is calculated to be 9.62 m^2^g^−1^, which is larger than that of commercial Pd black (6.88 m^2^g^−1^). The larger ECSA for the Pd–Ag alloy polyhedrons is most likely due to the smaller size and better dispersion of the Pd–Ag alloy polyhedrons than commercial Pd black. Meanwhile, it is observed that the formation potential of oxide on the Pd–Ag alloy polyhedrons negatively shifts ca. 35 mV compared to that of Pd black, indicating the Pd–Ag alloy polyhedrons can afford –OH species at lower potential.

The electrocatalytic properties of the Pd–Ag alloy polyhedrons and commercial Pd black for the FAOR are also investigated by cyclic voltammetry in acidic electrolyte, where the current were normalized with respect to Pd metal mass (Fig. 5B). In the positive potential scan direction, the peak current of the FAOR on the Pd–Ag alloy polyhedrons is measured to be ca. 287.7 Ag^−1^, which is 2.66 times greater than that of the commercial Pd black (108.1 Ag^−1^). Meanwhile, the onset oxidation potential of the FAOR on the Pd–Ag alloy polyhedrons shifts negatively ca. 30 mV compared to that of commercial Pd black. Such the higher oxidation peak current and the lower onset oxidation potential indicate that the Pd–Ag alloy polyhedrons have higher mass activity than that of the commercial Pd black, indicating the Pd–Ag alloy polyhedrons hold promise as a potential practical electrocatalyst for the FAOR. The Tafel curves of the FAOR on the Pd–Ag alloy polyhedrons and commercial Pd black keep consistent with linear relationship in electrochemical control region (Fig. 5C). However, the Pd–Ag alloy polyhedrons have a higher output current compared to Pd black under the same potential. Moreover, the polarization over-potential on commercial Pd black occurs at lower output current with respect to the Pd–Ag alloy polyhedrons, demonstrating the tremendously improved kinetics of FAOR on the Pd–Ag alloy polyhedrons. The linear portions of the Tafel plots are fitted to the Tafel equation (η = a + b log j, where η, a, b, and j are the overpotential, the intercept, the Tafel slope, and the current density, respectively). The exchange current density (j_o_) of FAOR on the Pd–Ag alloy polyhedrons is calculated to be 63.1 A g^−1^, which is higher than that for the Pd black (25.1 A g^−1^). According to the equation (R_ct_ = RT/j_o_F, where R, T and F are gas constant, absolute temperature and Faradic constant, respectively), the Pd–Ag alloy polyhedrons has lower charge transfer resistance (R_ct_) than that of the Pd black, indicating that the presence of Ag in the Pd–Ag alloy electrocatalysts assists in the effective charge transfer. Compared to the cyclic voltammogram of Pd–Ag alloy polyhedrons in H_2_SO_4_ solution without HCOOH, the cyclic voltammogram of Pd–Ag alloy polyhedrons in H_2_SO_4_ + HCOOH mixture solution shows a slightly enhanced hydrogen desorption peak at ca. −0.15 V potential (Figure S7), which can be ascribed to electrooxidation of H_2_ that released from formic acid decomposition.

Since the specific activity could effectively evaluate the intrinsic activity of electrocatalysts, the specific activities of the Pd–Ag alloy polyhedrons and Pd black were calculated through the normalization by the ECSA. As observed, the specific activity of the Pd–Ag alloy polyhedrons for the FAOR is also remarkably enhanced compared to that of the Pd black (Fig. 5D). For example, the oxidation current activity at 0.15 V (a typical working potential for the FAOR) are 30.1 and 15.6 Am^−2^ on the Pd–Ag alloy polyhedrons and commercial Pd black, respectively. The enhanced electrocatalytic activity of the Pd–Ag alloy polyhedrons is further confirmed by the turnover frequency (TOF, defined here as the HCOOH conversion per surface Pd atom per second) analysis based on the specific activity^34^,^35^. The TOF of the FAOR on the Pd–Ag alloy polyhedrons (6.44 atom^−1^ s^−1^) is 1.69 times bigger than that on Pd black (3.79 atom^−1^ s^−1^) at 0.15 V potential (Fig. 5E). In addition, the Pd–Ag alloy polyhedrons also show the improved stability for the FAOR, as indicated by chronoamperometry curves for 4000 s (Fig. 5F). Compared to commercial Pd black, the onset oxidation potential and peak potential of CO_ads_ on the Pd–Ag alloy polyhedrons obviously shift negatively (Figure S8). This result demonstrates that the Pd–Ag alloy polyhedrons have an improved CO_ads_ intermediate tolerance ability for the FAOR. Mainly, the alloying Pd with Ag facilitates removal of CO by activating H_2_O at the lower potential or accelerates the oxidation of poisonous –CO_ads_ intermediates.

## Discussion

In the absence of PdCl_2_, the Ag^+^ ions (*i.e.*, AgCl precipitate) can not be reduced by HCHO under the present experimental conditions, indicating that the reduction of the Ag^+^ precursor is facilitated by the preformed Pd crystal nuclei through the epiphytic reduction mechanism, in which the underpotential deposition (UPD) of Ag^+^ on Pd surface provides a good implementation platform^36^. Based on these experimental observation and discussion, the growth mechanism of the Pd–Ag alloy polyhedrons can be expressed as follows: Step i) the nucleation and growth of the AgCl nanocrystals due to the interaction between Ag^+^ and Cl^–^ at the initial low temperature reaction stage; Step ii) the liberation of Ag^+^ ion from AgCl and the nucleation of Pd crystal nuclei due to HCHO reduction at high temperature reaction stage; Step iii) the formation of initial Pd–Ag intermediate due to the catalytic reduction of the Ag^+^ by the performed Pd nuclei via the UPD pathway^36^,^37^; Step iv) the deposition growth of Pd atoms on the Pd–Ag intermediate due to HCHO reduction; Step v) the deposition growth of Ag atoms on the new generated Pd–Ag surface at Step iv due to the repeated catalytic reduction of the Ag^+^ ions by Pd atoms. In this way, the Pd^2+^ and Ag^+^ precursors are reduced in tandem to form the Pd–Ag alloy polyhedrons with a uniform composition. The synthesis method can also be used to prepare the other PdAg alloy nanoparticles with different atomic ratio. For example, Pd_3_Ag_1_ and Pd_1_Ag_3_ alloy nanoparticles can be synthesized by tuning the atom ratio of Pd/Ag (Figure S9). Compared to as-prepared Pd–Ag alloy polyhedrons (the ratio of Pd/Ag was approximately 1:1), the morphologies of Pd_3_Ag_1_ and Pd_1_Ag_3_ alloy nanoparticles have not obvious change, but their particle sizes are not uniform.

All mentioned electrochemical results indicate that the Pd–Ag alloy polyhedrons exhibit an enhanced catalytic electroactivity and durability for the FAOR compared to Pd black. It is clear that the electrocatalytic activity of Pd-based nanocrystals for the FAOR is highly relative to their electronic structure, chemical composition, and surface structure. The negative shift of Pd 3d binding energy on the Pd–Ag alloy polyhedrons relative to the standard value of bulk Pd and commercial Pd black (Figure S10) demonstrates that the electron density of Pd atoms from Pd–Ag alloy polyhedrons increases, which enhances the electron donation from Pd to the π* orbital of the poisonous intermediates. The increased electron donation effectively increases the bonding strength of reaction species on Pd surface, and consequently promotes the reaction kinetics of the FAOR. Meanwhile, Ag is more active than Pd at the low potential, which accelerates the oxidation of poisonous –CO_ads_ intermediates and consequently inhibit the poisoning of the Pd active sites based on bi-functional mechanism^12^. Meanwhile, the composition-dependent electrocatalytic activity for the FAOR is also investigated thoroughly. The catalytic activities for the FAOR follow the sequence of Pd_1_Ag_1_ ≈ Pd_3_Ag_1_ > Pd_1_Ag_3_ (Figure S11). Considering the high cost of noble metal Pd, the as-prepared Pd_1_Ag_1_ alloy polyhedrons may have better potential application than Pd_3_Ag_1_ nanoparticles.

In summary, we demonstrate a simple and efficient strategy for the synthesis of the Pd–Ag alloy polyhedrons with uniform size. Through the time sequential evolution experiments and a series of controlled experiments, the possible formation mechanism of Pd–Ag alloy polyhedrons is presented. Although the interaction between Ag^+^ and Cl^–^ results in the generation of AgCl precipitate, Ag^+^ ions slowly liberated from AgCl at high temperature, which can be catalytically reduced by newly formed Pd crystal nuclei and consequently transfer into the Pd lattice to form the Pd–Ag alloy. In addition, we found remarkably improved electrocatalytic activity and stability of the Pd–Ag alloy polyhedrons for the FAOR because of the unique polyhedral structure as well as the “synergistic effects” between Pd and Ag atoms, including electronic effect and bi-functional mechanism. Thus, it is expected that the Pd–Ag alloy polyhedrons can be utilized as a promising anodic electrocatalysts in DFAFCs applications.

## Methods

### Fabrication of Pd–Ag alloy polyhedrons

In a typical synthesis, 0.5 mL of 0.05 M PdCl_2_, 0.5 mL of 0.05 M AgNO_3_ and 1.0 mL of 0.50 M PAH (molarity of PAH given with respect to the repeating unit) aqueous solutions were added into 7.5 mL of water with continued stirring (Noting: the interaction between Ag^+^ and Cl^–^ generates AgCl precipitate during mixing). After adding a 0.5 mL of HCHO solution (40%), the resultant mixture (pH 7.0) was transferred to a 20-mL Teflon-lined stainless-steel autoclave, and was then heated at 180 °C for 2 h. After being cooled to room temperature, the obtained Pd-Ag alloy polyhedrons were separated by centrifugation at 15000 rpm for 15 min, washed with acetic acid for 12 h, and then dried at 60 °C for 5 h in a vacuum dryer.

### Characterization

Transmission electron microscopy (TEM) images and energy-dispersive X-ray spectroscopy (EDX) elemental mapping patterns were taken using a JEOL JEM-2100F transmission electron microscopy operated at 200 kV. The samples were prepared by placing a drop of the colloidal solution or catalyst powder dispersion in ethanol solution (99%) on a carbon film coated Cu grid (3 mm, 300 mesh), followed by drying under ambient conditions. X-ray diffraction (XRD) patterns were obtained on a Model D/max-rC X-ray diffractometer using Cu Ka radiation source (λ = 1.5406 Å) and operating at 40 kV and 100 mA. High-resolution X-ray photoelectron spectroscopy (XPS) was carried out on a Thermo VG Scientific ESCALAB 250 spectrometer with an Al Kα radiator. The binding energy was calibrated by means of the C 1s peak energy of 284.6 eV.

### Electrochemical tests

All electrochemical experiments were performed by using a CHI 660 C electrochemical analyzer. A standard three-electrode system was used for all electrochemical experiments, which consisted of a platinum wire as the auxiliary electrode, a saturated calomel reference electrode (SCE) protected by Luggin capillary with KCl solution as the reference electrode, and a catalyst modified glassy carbon electrode as the working electrode. Potentials in this study were reported with respect to SCE. All electrochemical measurements were carried out at 30 ± 1 °C.

For the preparation of the working electrodes, an evenly distributed suspension of catalyst was prepared by ultrasonic the mixture of 10 mg catalyst and 5 mL H_2_O for 30 min, and 6 μL of the resulting suspension was drop-cast onto the surface of the glassy carbon electrode (3 mm diameter). After drying at room temperature, 3 μL of Nafion solution (5 wt.%) was covered on the catalyst modified electrode surface and allowed drying again. Thus, the working electrode was obtained, and the specific loading of metal on the electrode surface was about 170 μg cm^–2^. Electrochemical measurements were conducted in N_2_-saturated 0.5 M H_2_SO_4_ solution or N_2_-saturated 0.5 M H_2_SO_4_ solution with 0.5 M HCOOH. The ECSA of Pd catalysts was calculated from the following equation (ECSA = Q/mC, m was the loading amount of Pd metal) by integrating the reduction charge (Q) of surface Pd(OH)_2_ and assuming a value of 420 μC cm^−2^ (C) for the reduction charge of a Pd(OH)_2_ monolayer on the Pd surface because ECSA of Pd catalysts could not be precisely assessed by coulometry in the “hydrogen region” due to the interference of hydrogen absorption in bulk Pd.

## Additional Information

**How to cite this article**: Fu, G.-T. *et al.* Polyhedral Palladium–Silver Alloy Nanocrystals as Highly Active and Stable Electrocatalysts for the Formic Acid Oxidation Reaction. *Sci. Rep.* **5**, 13703; doi: 10.1038/srep13703 (2015).

## Supplementary Material

Supplementary Information

## References

[b1] Rice C. *et al.* Direct formic acid fuel cells. J. power sources 111, 83–89 (2002).

[b2] Zhu Y., Ha S. Y. & Masel R. I. High power density direct formic acid fuel cells. J. power sources 130, 8–14 (2004).

[b3] Mrozek M. F., Luo H. & Weaver M. J. Formic acid electrooxidation on platinum-group metals: Is adsorbed carbon monoxide solely a catalytic poison? Langmuir 16, 8463–8469 (2000).

[b4] Rice C., Ha S., Masel R. & Wieckowski A. Catalysts for direct formic acid fuel cells. J. power sources 115, 229–235 (2003).

[b5] Xia B. Y., Wu H. B., Yan Y., Wang H. B. & Wang X. One‐Pot Synthesis of Platinum Nanocubes on Reduced Graphene Oxide with Enhanced Electrocatalytic Activity. Small 10, 2336–2339 (2014). 24610604 10.1002/smll.201302648

[b6] Yu X. & Pickup P. G. Recent advances in direct formic acid fuel cells (DFAFC). J. power sources 182, 124–132 (2008).

[b7] Chang J., Feng L., Liu C., Xing W. & Hu X. An Effective Pd–Ni2P/C Anode Catalyst for Direct Formic Acid Fuel Cells. Angew. Chem. Int. Edit. 53, 122–126 (2014). 10.1002/anie.201308620 24511636

[b8] Wu D., Cao M. & Cao R. Ru-assisted synthesis of {111}-faceted Pd truncated bipyramids: a highly reactive, stable and restorable catalyst for formic acid oxidation. Chem. Commun. 50, 12970–12972 (2014). 10.1039/c4cc05855k 25220420

[b9] Mazumder V. & Sun S. Oleylamine-Mediated Synthesis of Pd Nanoparticles for Catalytic Formic Acid Oxidation. J. Am. Chem. Soc. 131, 4588–4589 (2009). 19281236 10.1021/ja9004915

[b10] Ho S. F. *et al.* A facile route to monodisperse MPd (M = Co or Cu) alloy nanoparticles and their catalysis for electrooxidation of formic acid. Nanoscale 6, 6970–6973 (2014). 24838646 10.1039/c4nr01107d

[b11] Antolini E. Palladium in fuel cell catalysis. Energy Environ. Sci. 2, 915–931 (2009).

[b12] Zhang L. *et al.* Facile syntheses and electrocatalytic properties of porous Pd and its alloy nanospheres. J. Mater. Chem. 21, 9620–9625 (2011).

[b13] Fu G.-T. *et al.* Trimetallic PtAgCu@PtCu core@shell concave nanooctahedrons with enhanced activity for formic acid oxidation reaction. Nano Energy 12, 824–832, (2015).

[b14] Zhang L. *et al.* Crystalline palladium–cobalt alloy nanoassemblies with enhanced activity and stability for the formic acid oxidation reaction. Appl. Catal. B: Environ. 138, 229–235 (2013).

[b15] Hu S., Scudiero L. & Ha S. Electronic effect of Pd-transition metal bimetallic surfaces toward formic acid electrochemical oxidation. Electrochem. Commun. 38, 107–109 (2014).

[b16] Yin Z. *et al.* Monodispersed bimetallic PdAg nanoparticles with twinned structures: Formation and enhancement for the methanol oxidation. Sci. Rep. 4, 4288 (2014). 24608736 10.1038/srep04288 PMC3948072

[b17] Jiang H.-L., Akita T., Ishida T., Haruta M. & Xu Q. Synergistic Catalysis of Au@Ag Core–Shell Nanoparticles Stabilized on Metal–Organic Framework. J. Am. Chem. Soc. 133, 1304 (2011). 21214205 10.1021/ja1099006

[b18] Hammer B. & Nørskov J. K. Theoretical surface science and catalysis—calculations and concepts. Adv. catal. 45, 71–129 (2000).

[b19] Smith W. F. Principles of materials science and engineering. (McGraw Hill Book Co., 1986).

[b20] Chen J. *et al.* Optical properties of Pd-Ag and Pt-Ag nanoboxes synthesized via galvanic replacement reactions. Nano Lett. 5, 2058–2062 (2005). 16218737 10.1021/nl051652u

[b21] Tsuji M. *et al.* Synthesis of Ag–Au and Ag–Pd alloy triangular hollow nanoframes by galvanic replacement reactions without and with post-treatment using NaCl in an aqueous solution. CrystEngComm 16, 2684–2691 (2014).

[b22] Huang J. F., Vongehr S., Tang S. C. & Meng X. K. Highly Catalytic Pd-Ag Bimetallic Dendrites. J. Phys. Chem. C 114, 15005–15010 (2010).

[b23] Lu Y. & Chen W. Nanoneedle-covered Pd– Ag nanotubes: high electrocatalytic activity for formic acid oxidation. J. Phys. Chem. C 114, 21190–21200 (2010).

[b24] Abbasi N., Shahbazi P. & Kiani A. Electrocatalytic oxidation of ethanol at Pd/Ag nanodendrites prepared via low support electrodeposition and galvanic replacement. J. Mater. Chem. A 1, 9966–9972 (2013).

[b25] Xu L. *et al.* Triangular Ag–Pd alloy nanoprisms: rational synthesis with high-efficiency for electrocatalytic oxygen reduction. Nanoscale 6, 11738–11743 (2014). 25155648 10.1039/c4nr03600j

[b26] Chen Y., Zhu Q. L., Tsumori N. & Xu Q. Immobilizing Highly Catalytically Active Noble Metal Nanoparticles on Reduced Graphene Oxide: A Non-Noble Metal Sacrificial Approach. J. Am. Chem. Soc. 137, 106–109 (2015). 25543717 10.1021/ja511511q

[b27] Arblaster J. W. Crystallographic properties of palladium. Platin. Met. Rev. 56, 181–189 (2012).

[b28] Mikheev G. *et al.* Effect of the burning temperature on the phase composition, photovoltaic response, and electrical properties of Ag/Pd resistive films. Phys. Solid State 56, 2286–2293 (2014).

[b29] Li G., Jiang L., Jiang Q., Wang S. & Sun G. Preparation and characterization of Pd x Ag y/C electrocatalysts for ethanol electrooxidation reaction in alkaline media. Electrochim. Acta 56, 7703–7711 (2011).

[b30] Fu G. *et al.* Polyallylamine Functionalized Palladium Icosahedra: One-Pot Water-Based Synthesis and Their Superior Electrocatalytic Activity and Ethanol Tolerant Ability in Alkaline Media. Langmuir 29, 4413–4420 (2013). 23480348 10.1021/la304881m

[b31] Fu G. *et al.* One-Pot Water-Based Synthesis of Pt–Pd Alloy Nanoflowers and Their Superior Electrocatalytic Activity for the Oxygen Reduction Reaction and Remarkable Methanol-Tolerant Ability in Acid Media. J. Phys. Chem. C 117, 9826–9834 (2013).

[b32] Fu G. *et al.* Facile water-based synthesis and catalytic properties of platinum-gold alloy nanocubes. CrystEngComm 16, 1606–1610 (2014).

[b33] Zhang W., Yang J. & Lu X. Tailoring Galvanic Replacement Reaction for the Preparation of Pt/Ag Bimetallic Hollow Nanostructures with Controlled Number of Voids. ACS nano 6, 7397–7405 (2012). 22804563 10.1021/nn302590k

[b34] Jin M. S., Zhang H., Xie Z. X. & Xia Y. N. Palladium Concave Nanocubes with High-Index Facets and Their Enhanced Catalytic Properties. Angew. Chem. Int. Edit. 50, 7850–7854 (2011). 10.1002/anie.201103002 21732512

[b35] Zhao R. *et al.* Multi-generation overgrowth induced synthesis of three-dimensional highly branched palladium tetrapods and their electrocatalytic activity for formic acid oxidation. Nanoscale 6, 2776–2781 (2014). 24463486 10.1039/c3nr05718f

[b36] Yu Y. *et al.* Learning from nature: introducing an epiphyte–host relationship in the synthesis of alloy nanoparticles by co-reduction methods. Chem. Commun. 50, 9765–9768 (2014). 10.1039/c4cc04132a 25025323

[b37] Soliman K. & Kibler L. Variation of the potential of zero charge for a silver monolayer deposited onto various noble metal single crystal surfaces. Electrochim. Acta 52, 5654–5658 (2007).

