# Supplementary material for: Polyhedral Palladium–Silver Alloy Nanocrystals as Highly Active and Stable Electrocatalysts for the Formic Acid Oxidation Reaction
Source: Sci Rep. 2015 Sep 2;5:13703. doi: 10.1038/srep13703 (PMC4556982; doi:10.1038/srep13703)
Supplement: Supplementary Information [file srep13703-s1.doc]

Supporting Information

Polyhedral Palladium–Silver Alloy Nanocrystals as Highly Active and Stable Electrocatalysts for the Formic Acid Oxidation Reaction

Geng-Tao Fu^1^, Chang Liu^1^, Qi Zhang^2^, Yu Chen*^2^, & Ya-Wen Tang*^1^

^1^ Jiangsu Key Laboratory of New Power Batteries, Jiangsu Collaborative Innovation Center of Biomedical Functional Materials, School of Chemistry and Materials Science, Nanjing Normal University, Nanjing 210023, P. R. China; ^2^ Key Laboratory of Macromolecular Science of Shaanxi Province, School of Materials Science and Engineering, Shaanxi Normal University, Xi'an 710062, P. R. China.

*Corresponding authors. Tel.: +86–25–85891651; fax: +86–25–83243286.

*E-mail address*: ndchenyu@gmail.com (Y. Chen); tangyawen@njnu.edu.cn (Y. Tang)

**Figures**

**Scheme S1.** The structure of the polyallylamine hydrochloride (PAH).

**Figure S1.** XPS survey scan spectrum of the Pd-Ag polyhedrons.


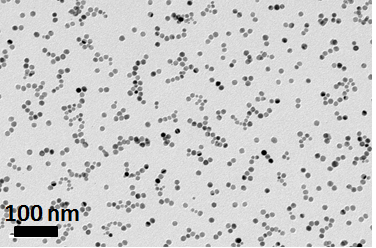


**Figure S2.** Large-area TEM image of as-prepared Pd-Ag polyhedrons.


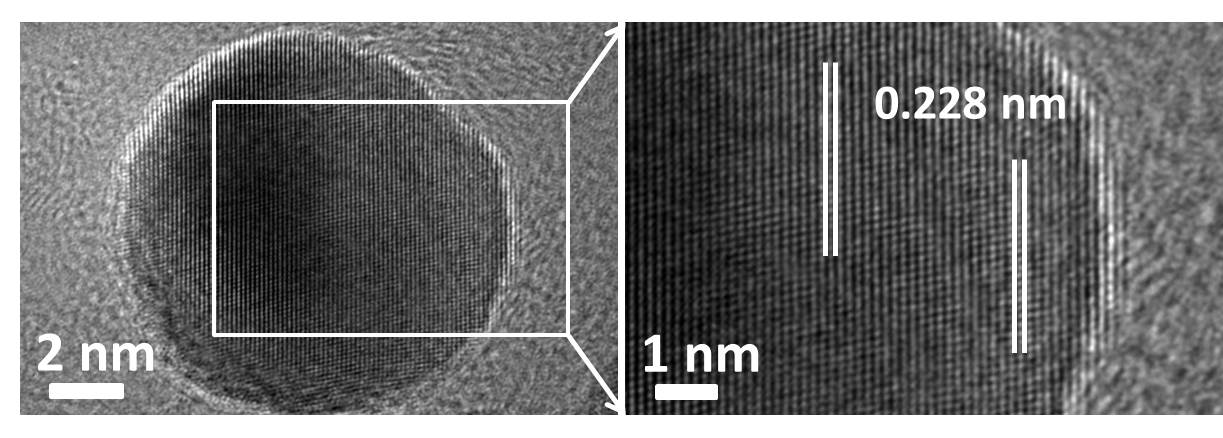


**Figure S3.** Magnified HRTEM image of an individual Pd-Ag polyhedrons.


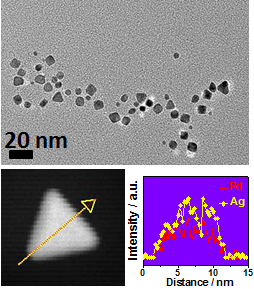


**Figure S4.** TEM image, HAADF-STEM image and EDX line scanning profiles of the intermediates collected at 30 min.


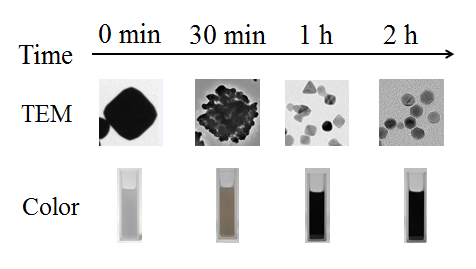


**Figure S5.** TEM images and digital photographs of the Pd-Ag the intermediates at different growth stages.


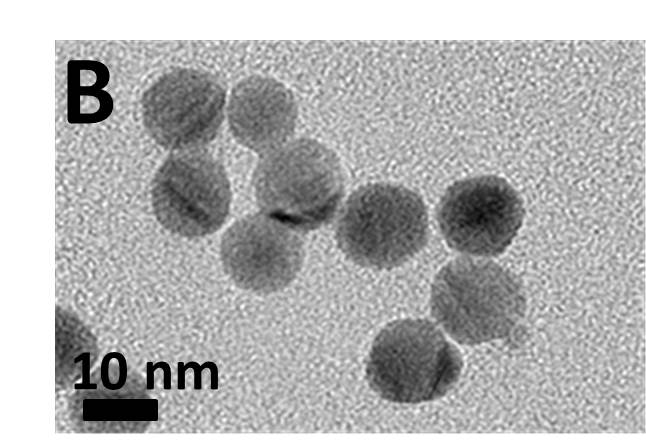


**Figure S6**. (A) EDX spectrum and (B) TEM image of the Pd–Ag alloy polyhedrons after an additional 10 CV cycles.


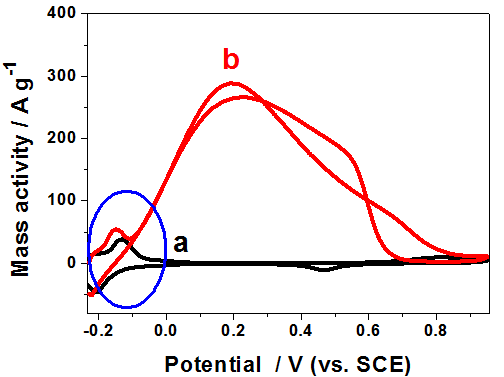


**Figure S7**. Cyclic voltammograms of the Pd–Ag alloy polyhedrons in (a) N_2_-saturated 0.5 M H_2_SO_4_ and (b) 0.5 M H_2_SO_4_ + 0.5 M HCOOH solutions at a scan rate of 50 mV s^−1^.

**Figure S8**. CO-stripping voltammograms of (a) Pd–Ag alloy polyhedrons and (b) Pd black after the stability measurement in 0.5 M H_2_SO_4_ solution at the scan rate of 50 mV s^−1^.


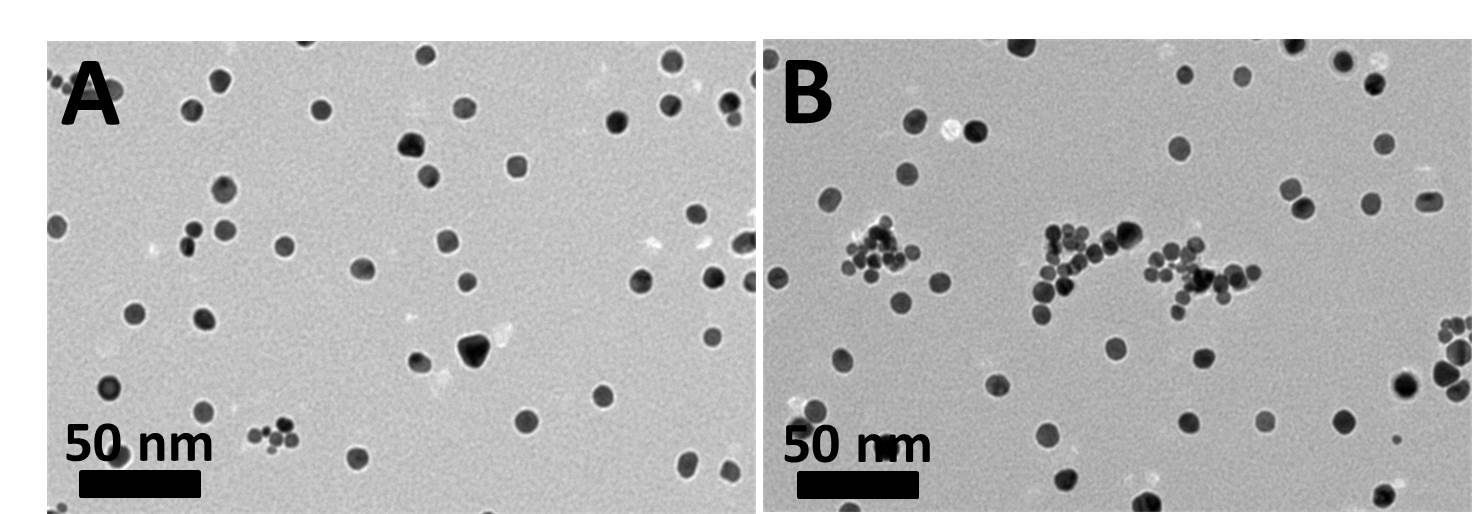


**Figure S9**. TEM images of Pd–Ag alloy nanoparticles with the different atom ratios of Pd/Ag: (A) 3:1 and (B) 1:3.

**Figure S10.** XPS spectra of (a) commercial Pd black and (b) Pd-Ag polyhedrons in the Pd 3d region.

**Figure S11.** Cyclic voltammograms of the Pd_1_Ag_1_, Pd_3_Ag_1_ and Pd_1_Ag_3_ in N_2_-saturated 0.5 M H_2_SO_4_ + 0.5 M HCOOH solution at a scan rate of 50 mV s^−1^.
